# Current Concepts and Management Approaches in Nonalcoholic Fatty Liver Disease

**DOI:** 10.1155/2013/481893

**Published:** 2013-03-20

**Authors:** Bashar M. Attar, David H. Van Thiel

**Affiliations:** ^1^Division of Gastroenterology and Hepatology, Cook County Health and Hospitals System, John H. Stroger Hospital of Cook County, Rush University Medical Center, 1901 West Harrison Street, Administration Building, Suite 1450, Chicago, IL 60612, USA; ^2^Rush Oak Park Hospital, Oak Park, IL 60642, USA

## Abstract

Nonalcoholic fatty liver disease (NAFLD) is the most common cause of liver dysfunction worldwide. NAFLD may progress to nonalcoholic steatohepatitis (NASH) and in turn cirrhosis. Importantly, hepatic cancer can occur in NASH in the absence of cirrhosis. The cardinal histologic feature of NAFLD is the presence of an excessive accumulation of triacylglycerols and diacylglycerols in hepatocytes. The presence of obesity and insulin resistance lead to an increased hepatic-free fatty acid (FFA) flux creating an environment appropriate for the development of NAFLD. The generation of toxic reactive oxygen species with the production of hepatic injury and inflammation as a consequence of FFA oxidation will ultimately lead to the initiation and progression of fibrosis. Lifestyle modifications specifically weight loss, physical exercise, and cognitive behavior therapy have been recommended as treatments for NASH. Dietary fructose is an independent risk factor for the development of NAFLD. Pioglitazone can be used to treat biopsy-proven NASH; however, its safety risks should be considered carefully. Greater consumption for coffee, independent of its caffeine component, has been associated with a significant reduced risk of advanced fibrosis in NASH. Additional data are needed before recommending bariatric surgery as an established option for the specific treatment of NASH.

## 1. NAFLD: What Is the Problem?

 Nonalcoholic fatty liver disease (NAFLD) is the most common cause of liver dysfunction worldwide. It is a spectrum of liver disease that ranges from simple fatty infiltration of the liver parenchyma (steatosis) to fat with inflammation (nonalcoholic steatohepatitis; NASH) and ultimately cirrhosis occurring in absence of excessive alcohol consumption defined as an upper threshold of 30 g/day for men and 20 g/day for women [1]. Histologic steatosis is defined as an increase in hepatocyte fat content. Hepatocellular ballooning, lobular inflammation, with or without acidophilic bodies, spotty necrosis, and perisinusoidal fibrosis are the major histologic manifestations of NASH.

The prevalence of NAFLD in the general population of developed countries is estimated at 25–30%. It is highest in populations with preexisting metabolic conditions such as obesity and type II diabetes. The obesity epidemic has led to a major increase in the prevalence of NAFLD. Liver biopsies, performed on US healthy potential live liver donors, revealed that 20% of potential live liver donors had a degree of steatosis beyond 30% [2]. The prevalence of NAFLD in selected populations of normal weight individuals without confounding metabolic risk factors is reported to be 16% [3]. In contrast, the prevalence of biopsy-proven NASH in the general population is estimated to be 3–5% [4]. Williams et al. evaluated 328 individuals who underwent right upper quadrant ultrasound exams with subsequent liver biopsy if ultrasound suggested the presence of hepatic steatosis [5]. The prevalence of NAFLD in this study was 46% which is higher than other reported estimates. Hispanics have the highest prevalence of NAFLD (58%) followed by Caucasians (44%) and African Americans (35%). NASH accounted for 12.2% of the total cohort and 29.9% of the ultrasound-positive patients [5]. Male patients had significantly higher rates of NAFLD (58.9%) and NASH (65%). Limitations of this study included a possible selection bias.

The natural history of NAFLD is not entirely known. NAFLD progresses in approximately 20% of cases to NASH and in turn cirrhosis in 20% of the cases with NASH. What is known and disturbing is that hepatic cancer can occur in NASH in the absence of cirrhosis [6]. Additionally, it is now believed that patients prone to NAFLD may go straight to a NASH phenotype and not progress from simple steatosis to NASH. Patients with NASH have a reduced survival due to liver-related deaths and its associated comorbidities that include cardiovascular disease, diabetes mellitus, hypertension, and sleep apnea.

## 2. Demographical and Clinical Factors

 Older age is an independent risk factor for hepatic steatosis [7]. Older individuals not only have a higher prevalence of NAFLD but also a greater likelihood of mortality and disease progression to fibrosis, type II diabetes mellitus, and hepatocellular carcinoma [8–11]. 

However, the association between increased age and the greater prevalence of NAFLD, and the progression of fibrosis with the development of cirrhosis with age may be a result of the longer duration of disease occurring in an older individual rather than age per se. Specifically, Hui et al. have reported that age does not differ between individuals whose NAFLD has progressed to NASH or cirrhosis as compared to those who did not [12]. The male gender has been recognized also as a risk factor for NASH, hepatic fibrosis, hepatic cancer, and an overall increased mortality [9, 13]. 

 A recent study that reviewed Medicare patients considered NAFLD as the third most common risk factor for hepatocellular cancer (HCC) accounting for 16% compared with history of prior hepatic infection (44%) or alcoholic liver disease (19%) [6]. These findings were based on a review of 17,895 HCC cases in the Surveillance, Epidemiology, and End Results (SEER) Medicare database. Cirrhosis was not present in 36% of the NAFLD-related HCC cases. Interestingly, 18% of the latter group had only steatosis and not NASH. These findings may explain the significant increase of the average number of cases reported per year of NAFLD-related HCC without cirrhosis from 51 in 1993–2000 to 88 in 2001–2007 as compared with no change in cases reported with cirrhosis [6]. This study however has several limitations including the determination of the level of accuracy regarding the presence of NASH or cirrhosis in the study population and the absence of a systematic centralized assessment of liver histopathology.

**Figure 1:**
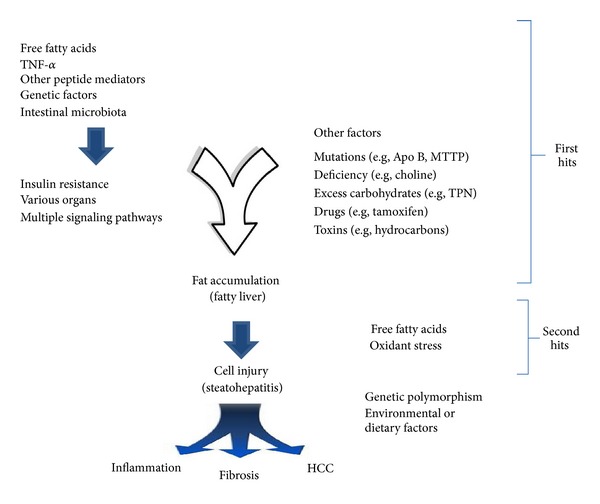
Postulated mechanisms of fat-induced inflammation and fibrosis in NAFLD and NASH. Modified from [79].

 Several studies have reported that within ethnic groups, Hispanics have the highest prevalence of NAFLD, hepatic steatosis, and elevated aminotransferase levels, followed by non-Hispanic Whites, while African Americans have the lowest rate of NAFLD [5, 14]. The explanation for these ethnic differences in the prevalence of NAFLD is unclear but probably includes both genetic and environmental factors (Figure 1).

## 3. Pathogenesis

The cardinal histologic feature of NAFLD is the presence of an excessive accumulation of triacylglycerols (TAG) and diacylglycerols (DAG) in hepatocytes. The accumulating hepatic TAG consists predominantly of DAG which has been hydrolyzed from TAG and is only reesterified back to TAG just before being secreted into the blood stream [15]. The presence of obesity and insulin resistance lead to an increased hepatic-free fatty acid (FFA) flux creating an environment appropriate for the development of NAFLD/NASH. The resultant net increase in hepatic FFA is hepatotoxic unless it is converted to nontoxic intracellular triglyceride (TG). When the synthesis of TG is impaired, the levels of FFA in the liver is increased with subsequent increase in hepatic fatty acid oxidation resulting in an increase in the overproduction of reactive oxygen species (ROS) also known as free radicals causing hepatocellular injury [16].

Based on this biochemical knowledge, a two-hit hypothesis for the pathogenesis of NASH has been proposed (Figure 1). The first hit involves the accumulation of excess triglyceride and particularly FFA in hepatocytes. The second hit is the generation of toxic reactive oxygen species with the production of hepatic injury and inflammation as a consequence of FFA oxidation which ultimately leads to the initiation and progression of fibrosis [17]. Evidence suggests that the accumulation of lipid droplets within hepatocytes from adipocytes is a consequence of an excessive exposure of hepatocytes to free fatty acids and represents a form of “ectopic fatty acid storage.” The subsequent increased hepatic content of triglycerides as opposed to FFAs may actually be a protective response that reduces the pathogenic potential of FFA within liver cells [18, 19]. Current data suggests that the intracellular molecular events involved in hepatic metabolism of fat (TG, DAG, and FFA) are the triggers for NAFLD and NASH. Specifically, as a result of increased *β*- and *ω*-FFA oxidation of fatty acids, an intracellular oxidative stress occurs due to the generation of reactive oxygen sepsis (ROS). The ROS in turn activates cytokine release by hepatocytes as well as the other cells contained in the liver to include myofibroblasts, endothelial cells, fibroblasts, and so forth, all of which can initiate various immune-mediated mechanisms leading to liver cell injury [20]. Moreover, biochemical evidence suggests that the changes in lipid partitioning within the liver are associated with an increase in hepatocyte apoptosis [21–24]. 

 The progression from simple hepatocellular steatosis to NASH is associated with the above-identified biochemical alterations occurring in concert with systemic hypertriglyceridemia and hyperbetalipoproteinemia. The development of a rapid weight loss due to any of many different reasons that include a requirement for central hyperalimentation on exposure to various hepatotoxins including medically prescribed drugs can enhance the progression of NAFLD to NASH. Thus, the current goals of NAFLD interventions are directed at a reduction of the flux of fatty acids through the liver, their excessive release from adipose tissue (lipolysis), and a reduction in both hepatic oxidation and de novo lipogenesis. The latter occurs as a consequence of an excessive metabolism of carbohydrates. Each of these processes is enhanced as a result of insulin resistance. Specifically, insulin resistance is associated with hyperinsulinemia, impaired glucose disposal by muscle, inappropriate glucose production by the liver, and enhanced adipocyte lipolysis as well as the promotion of de novo hepatic lipogenesis [25, 26]. 

 Why some patients with NAFLD present with a slow or rapid progression to NASH is poorly understood. Some of these patients may have insidious disease and present with hepatocellular carcinoma (HCC) or advanced NASH. Studies of hedgehog (Hh) signaling suggest that aberrant or prolonged Hh signaling during hepatic tissue repair may be an essential element for disease progression in NASH [27]. The level of Hh activation seems to be proportional to the severity and duration of liver injury in both rodents and humans.

 Specific genetic variations have been shown to play an important role in the pathogenesis of NAFLD and could potentially explain the interaction between the disease, ethnicity, environmental influences, other phenotypes, and potentially affect treatment strategies. It has been estimated that genetic factors influence the development of NAFLD in 26–35% of those with the disease. Consistent with this analysis is the fact that Schwimmer et al. documented a familial clustering of NAFLD. Additional studies investigating large family-based cohorts have estimated that the heritability factor for NAFLD is 0.27 [28]. Nevertheless, NAFLD is best considered a complex disease process wherein subtle interpatient genetic variations and environmental factors interact to determine both disease phenotype and progression [29].

 Romeo et al. identified one of the more significant genetic contributors to NAFLD. The missense rs738409 C/G single-nucleotide polymorphism (SNP) implying an amino acid change from isoleucine (I) to methionine (M) at the position 148 (I148 M) of the protein encoding the patatin-like phospholipase domain-containing 3 gene (PNPLA3), which is also known as adiponutrin, is associated with an increased hepatocyte fat content [30, 31]. Sookoian and Pirola have reported that the rs738409 CG allele is the most frequent gene variant present in individuals with NASH. In a study that included 2124 NAFLD individuals, the incidence of NASH was substantially increased in those who were GG homozygotes as compared to those with the normal CC genotype (OR 3.488, 95% confidence interval [CI] 1.859–6.545) [32]. The G allele has been shown to have a significant, unequivocal association with an increased risk of hepatic triglyceride accumulation and the finding of NAFLD [30, 31]. GG homozygotes have a 73% greater hepatic lipid content as compared to those having the normal wild-type CC genotype.

 The G allele is the more prevalent in Hispanic populations (0.49) as compared to others. This may explain why this ethnic group has the highest prevalence of NAFLD. In contrast, a substantially lower frequency of the G allele is observed in Caucasians (0.23) and African Americans (0.17). Another variant of the same gene, PNPLA3-S453I, which is observed more commonly in African Americans (0.104) but rarely in European Americans (0.003) and Hispanics (0.008), is associated with a significantly lower hepatic fat content and may be a NAFLD protective factor occurring in the African American population [32, 33]. 

The role of PNPLA3 variants and the degree of fat accumulation in the liver has been substantiated further in a large study of 7176 individuals assessing CT-detected hepatic steatosis. Importantly, 592 subjects in this study had biopsy-proven NAFLD [34]. Speliotes et al. investigated common index genetic variants in or near 5 genes that are associated with NAFLD in individuals of European ancestry [35]. The genetic variants examined included PNPLA3 (patatin-like phospholipase domain-containing protein 3), NCAN (neurocan), LYPLAL1 (lysophospholipase-like 1), GCKR (glucokinase regulatory protein), and PPP1R3B (protein phosphatase 1), regulatory subunit 3b. Together these variants were calculated to account for about 20% of the heritability of NAFLD [34, 35]. The GCKR variant has been identified independently as an NAFLD-associated gene in Chinese subjects [36]. Importantly, four of these genetic factors (all but PPP1R3B) are positively associated with NASH and fibrosis (OR > 1.37) [35]. 

 Other genetic factors potentially involved in the pathogenesis of NAFLD and its progression include HNF4A, a type 2 diabetes-associated gene which is important in lipid oxidations and various genetic variants of CYP4F2 which plays a role in predicting the response of individuals with NAFLD to the currently available therapies [37]. 

Recent data have linked both obesity and NAFLD to specific variations of the intestinal microbiota. Specifically, the intestinal microbiota (IM) composition has been shown to differ between obese and lean individuals. These studies suggest that specific intestinal bacteria may contribute to the pathogenesis of obesity and as a result be associated with NAFLD. Consistent with this line of thought, Mouzaki et al. have shown that the IM of patients with NASH is unique. They compared three groups: individuals with biopsy-proven nonalcoholic steatohepatitis (NASH), simple steatosis (SS), and healthy controls (HC) and compared their fecal content of Bifidobacteria, Bacteroidetes, *C. coccoides*, *C. leptum*, *E. coli*, total bacteria, and Archaea. As expected, the body mass index (BMI) was higher in NASH patients as compared to HC (*P* = 0.001) and the percentage of fat intake adjusted for the basal metabolic rate (BMR) was higher in HC compared to both the SS and NASH groups (*P* = 0.04). Moreover, the fecal level of Coccoides was greater in NASH patients as compared to the SS group (*P* = 0.04). The percentage of Bacteroidetes was lower in NASH patients (*P* = 0.027; CI: −1.71 to −0.11) as compared to both the SS and HC groups (*P* = 0.006). Importantly, the percentage of Bacteroidetes in the stool correlated negatively with the HOMA-IR score, a measure of insulin resistance, in patients with NAFLD (*r* = −0.49; *P* = 0.002) producing additional evidence of the benefit of IM enriched with Bacteroidetes species [38].

## 4. Diagnostic Approaches of NAFLD

 Individuals, who present with persistently abnormal AST, ALT, or Alkaline phosphatase levels; persistently unexplained hepatomegaly; or an abnormal hepatic imaging study consistent with increased fat content in the liver suggestive of NAFLD should be evaluated for the presence of NAFLD. AST and ALT levels can be modestly elevated or normal, although the ratio of AST to ALT is typically less than 1 in individuals with NAFLD. An elevated serum uric acid level is associated with a 1.29 hazard ratio of having NAFLD (20% are found to have hyperuricemia and many of these have clinical gout). The serum ferritin level is 1.5 times higher than the upper limit of normal in many individuals with NAFLD and importantly is associated with a more advanced histologic stage of the disease (NASH). 

**Figure 2:**
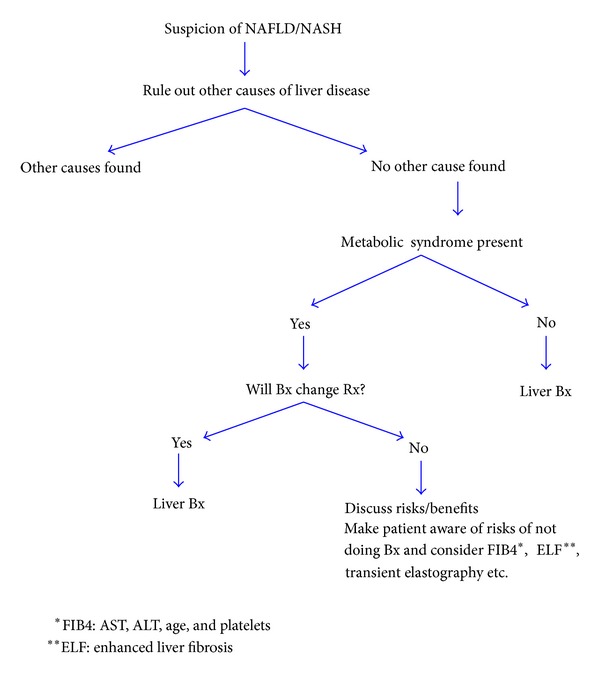
Diagnostic approaches to the patient suspected of having NAFLD/NASH [80].

Noninvasive radiological modalities such as ultrasonography, computed tomography (CT), and magnetic resonance imaging (MRI) can detect hepatic steatosis even in those with normal hepatic enzymes levels. Quantification of the severity of the hepatic steatosis can be accomplished with MRI techniques, although the sensitivity of the procedure currently is rather low. Liver stiffness, a surrogate marker for fibrosis, can be measured by transient elastography (fibroscan). The latter has been particularly helpful in identifying individuals with advanced fibrosis (stages 3 and 4) from those with lesser degree of fibrosis. However, increased liver stiffness can also be seen in the absence of fibrosis. Unfortunately, none of the available noninvasive blood tests for fibrosis or for that matter imaging procedures can distinguish simple steatosis from NASH. As a result, liver biopsy is the gold standard which is utilized to confirm a diagnosis of NAFLD/NASH (Figure 2). Liver biopsy also enables the grade of inflammation as well as the stage of fibrosis to be assessed. It also serves to exclude individuals with other histologically identifiable diagnoses that can be confused with those with NAFLD. Thus, it should be considered in all patients with NAFLD particularly those in clinical studies.

## 5. What Then Is the Appropriate Clinical Management of NAFLD?

Lifestyle modifications specifically weight loss, physical exercise, and cognitive behavior therapy have been recommended as treatments for nonalcoholic steatohepatitis (NASH). The rationale for this approach stems from complex factors identified as playing a role in insulin resistance and the resultant lipotoxicity of FA in the pathogenesis of NAFLD and its progression to NASH and promotion of fibrogenesis. Evidence exists to document that lower physical fitness is associated with an increased severity of nonalcoholic fatty liver disease (NAFLD) [39]. Conversely, increased physical activity is associated with reduced abdominal fat, reduced intrahepatic fat, and improved insulin sensitivity, all factors that are present in individuals with NAFLD and the metabolic syndrome [40, 41].

 Well-designed studies of exercise that eliminate confounding factors in the analysis, such as weight loss and dietary changes, are needed in NAFLD. Achieving a weight loss of at least 9% has been shown to improve steatosis and has a modest effect on hepatic inflammation but does not appear to reduce the stage of fibrosis [42]. Whether it reduces the progression of fibrosis has not yet been investigated.

 Other studies have demonstrated that a small 5–10% weight loss can lead to aminotransferase normalization consistent with a reduced level of hepatic injury [43, 44]. Weight loss of as little as 3–5% of body weight improves steatosis. Weight loss of 10% or more with dietary management and exercise appears to have an additional effect on improving the level of necroinflammation. The long-term consequences of such changes in weight loss on the progression of fibrosis remain to be determined in longitudinal studies.

 No weight-loss medication has been identified as yet to have long-term safety, efficacy, and tolerability. Individuals who achieve a modest weight loss with medication specific for weight loss typically regain their weight upon medication discontinuation. Orlistat, a lipase inhibitor, which has been approved by the Food and Drug Administration (FDA) for use up to 12 weeks has been shown to modestly reduce body weight but its use is complicated by several gastrointestinal side effects to include diarrhea, incontinence, abdominal gas, and flatus [45]. 

Behaviorally based weight-management programs focus on energy balance by increasing exercise, reducing calorie intake, restructuring maladaptive thinking patterns, and goal setting. These programs have shown limited success due to the high frequency of behavioral relapse with resultant weight gain after terminating the behavioral intervention program [46].

It has been estimated that approximately 2% of the general population manifests a “binge-eating disorder (BED)” which is associated frequently with an increased BMI. The presence of BED in weight-loss studies is associated with poorer weight-loss outcomes. Cognitive behavioral therapy (CBT) for BED is superior to behavioral weight management alone in achieving binge-eating behavior reduction. Weight-loss outcomes in CBT alone however are poorer than those seen in weight-management interventions [47].

Adherence to diet and particularly the composition of the diet are important factors in determining an improved outcome in individuals treated for NAFLD. The consumption of soft drinks and drinks with a high-fructose corn syrup content as well as intake of dietary *trans*-fats-enriched foods is associated with insulin resistance and the development of hepatic steatosis. The specific mechanisms responsible for the development of hepatic steatosis with these products are unclear but are thought to be a consequence of the hepatic metabolism of fructose favoring ATP depletion, lipotoxicity, and insulin resistance combined with an enhanced TNF expression [48, 49].

Consistent with this concept, several studies have implicated dietary fructose as an independent risk factor for the development of both NAFLD and the metabolic syndrome. In contrast to glucose, fructose ingestion does not result in leptin secretion and the subsequent sensation of satiety. Moreover, fructose ingestion increases de novo lipogenesis. Although more studies are needed, it is recommended that NAFLD patients should be advised currently to avoid high-fructose corn syrup and fructose-containing soft drinks and foods containing *trans*-fat [50, 51].

 A low *n*–3/*n*–6 polyunsaturated fatty acids (PUFA) dietary ratio has been identified in individuals with NASH [52–54]. Diets supplemented with PUFA have been shown to have a beneficial effect on both hepatic lipogenesis and steatosis [55]. Increasing the consumption of omega-3 polyunsaturated fatty acids results in improvements in hepatic lipid metabolism and reduces hepatic steatosis [53]. Additional studies are needed however before recommending omega-3 fatty acids for the specific treatment of either NAFLD or NASH.

## 6. Vitamin E

Vitamin E (*α*-tocopherol) has been utilized to treat NAFLD because it inhibits both oxidative stress acting as a free radical scavenger and reduces hepatic profibrotic activity. Several limited underpowered studies conducted using vitamin E at different doses have been shown to result in reductions in serum aminotransferases levels, an improvement in hepatic steatosis, inflammation, and cellular ballooning as well as the resolution of steatohepatitis in patients with NASH. No improvement in the stage of fibrosis has been reported to occur in these studies [56].

The PIVENS study is the largest study using a high dose of vitamin E (800 IU/d) for 96 weeks in nondiabetic patients with biopsy-proven NASH. It documented a significant reduction in hepatocellular inflammation and ballooning in hepatic biopsies with the use of vitamin E 42% versus 19% in placebo-treated individuals *P* < 0.001. Importantly, no significant adverse effects occurred during the trial [57]. The TONIC trial, conducted in children, resulted in improvements in aminotransferases levels and hepatic histology similar to the PIVENS [58].

Physicians, who recommend vitamin E, should consider the potential risks of vitamin E therapy. A meta-analysis of more than 135,000 patients taking vitamin E supplements (400 IU/day) demonstrated an increase in all-cause mortality consisting of an additional 39 deaths per 10,000 individuals. This increase in mortality was dose-dependent and began at 150 IU/d [59]. Moreover, the administration of vitamin E at a dose of 400 IU/day has been shown to significantly increase the risk of prostate cancer. The absolute increased risk for prostate cancer is 1.6 per 1000 person years of vitamin E use [60]. Awaiting further data, the recently published AASLD guidelines for the treatment of NAFLD/NASH do not recommend vitamin E therapy in diabetic patients, NAFLD individuals without a liver biopsy confirmation, NASH that has progressed to cirrhosis, or individuals with cryptogenic cirrhosis because of these risk concerns [61].

## 7. Insulin Sensitizers

### 7.1. Metformin

An open-label study comparing 110 patients with NASH, who received either metformin 2 grams/day, vitamin E 800 IU/day, or dietary-induced weight loss for 12 months, found a greater aminotransferase improvement occurred with the use of metformin as compared to vitamin E therapy or dietary management alone. In a small subset of these patients, who underwent liver biopsies before and after treatment, a modest histologic improvement in hepatocellular inflammation and steatosis was observed. No reduction in fibrosis was found [62].

A randomized controlled trial investigating the effect of metformin versus placebo failed to show an improvement in the aminotransferase levels or liver histology despite controlling for differences in diet and exercise interventions present in both arms of the study [63]. This failure to achieve a response was confirmed subsequently by a meta-analysis which demonstrated that lifestyle intervention plus metformin for 6–12 months, regardless of the metformin dose or the presence of diabetes, failed to produce an improvement in aminotransferase levels or hepatic histology [64]. Therefore, metformin is not recommended as a primary treatment for NASH [61]. 

### 7.2. Thiazolidinediones (TZDs)

Thiazolidinediones enhance insulin sensitivity by acting on peroxisome proliferator-activated receptor gamma and increasing circulating adiponectin [65]. Improving insulin sensitivity utilizing thiazolidinediones has been shown to prevent the activation of adipocyte c-jun kinase, a kinase that when activated impairs adipocyte responsiveness to insulin and adipocyte storage of TG [18]. Several studies including a meta-analysis that included 5 randomized controlled trials demonstrated that pioglitazone significantly improved aminotransferase levels, hepatic inflammation, and steatosis but did not alter the stage of fibrosis [64]. In contrast, a meta-analysis of 4 randomized, placebo-controlled clinical trials using TZDs in the treatment of NASH showed that pioglitazone significantly improved hepatic steatosis, hepatocellular ballooning degeneration, hepatic lobular inflammation, as well as fibrosis [66]. 

The PIVENS study, a large multicenter randomized controlled trial discussed above investigated 247 nondiabetic patients with NASH randomized for treatment either with pioglitazone (30 mg/day), vitamin E (800 IU/day), or placebo for 24 months. This study reported a statistical improvement in hepatocellular steatosis and ballooning but did not achieve a level of statistical significance for improvement in the stage of fibrosis [57]. 

There are concerns about the long-term use of TZDs which include weight gain (average 4 Kg), congestive heart failure (CHF), other cardiovascular morbidity, and bone loss with an increased fractures risk, as well as an increase in reported frequency of urinary bladder cancers. As a result, pioglitazone can be used to treat patients with biopsy-proven NASH especially with a more advanced stage of fibrosis that have not responded to adequate trial of aggressive lifestyle change. However, pioglitazone safety risks should be considered carefully [61].

## 8. Coffee

Coffee is a complex mixture of more than a 1,000 compounds with the major constituent being caffeine. The other two main components are diterpenes, such as cafestol and kahweol, and chlorogenic acids. Several studies have linked coffee consumption to an improvement in NAFLD [67, 68]. A recent study suggested that the serum aminotransferase levels in individuals suspected of having NAFLD are higher in those who consume lesser amounts of coffee [69]. A potential mechanism for this observation is that caffeine alters TGF*β* signaling pathways by increasing the level of SMAD, which reduces the transcription of CTGF, a major stimulator of fibrosis [69–71].

 Molloy et al. demonstrated an inverse relationship between regular coffee caffeine consumption and hepatic fibrosis in patients with NASH. Importantly, total caffeine was not significantly correlated with risk of NASH versus no NASH. Nevertheless, coffee intake significantly decreased risk of advanced fibrosis in NASH and showed a negative correlation with NASH fibrosis stage 2–4 versus NASH stage 0-1. These findings suggest that other properties of coffee beyond caffeine may affect disease progression in patients with NASH [72]. Additional studies have shown that coffee induces UDP glucuronosyltransferases, which may be responsible for coffee's protective antioxidant effect in individuals with liver disease independent of the caffeine, cafestol, or kahweol content of the beverage ingested [69]. Interestingly, another study which enrolled severely obese European individuals revealed that there is no association between the presence of biopsy-proven NASH and the consumption of coffee [73]. Moreover, the total consumption of coffee whether as regular coffee or espresso was not associated with NASH. On the other hand, regular coffee but not espresso has been identified as an independent protective factor in biopsy-proven NASH. This latter finding suggests that a component of coffee is responsible for the reduction of hepatic inflammation and fibrosis in morbidly obese patients with NAFLD or NASH. In contrast to regular coffee, this beneficial effect was not observed with espresso and was associated with the metabolic syndrome. All of these studies have limitations. These include but are not limited to a lack of information on the levels of caffeine and the specific coffee brewing methods utilized [73, 74]. It is of interest to know that a similar protective action of coffee against hepatic inflammation and fibrosis has been shown in patients with hepatitis C [75].

## 9. Bariatric Surgery

Bariatric surgery is effective in weight reduction (48–70%) and its effect varies according to the type of surgery performed being less with gastric banding and greater with bypass procedures [76]. A meta-analysis of several types of bariatric surgery demonstrated a significant improvement or complete resolution of steatosis in 92% of patients after surgery; 82% of patients showed an improvement or resolution of the histologic findings of NASH and 66% expressed an improvement in the stage of hepatic fibrosis [77]. These data, however, are based on observational studies and the conclusions are derived from studies with less rigorous inclusion criteria and without an assessment of the potential presence of biases induced by confounding factors. When evaluating these advantageous changes in fibrosis with bariatric surgery, it needs to be pointed out that in some cases the fibrosis progressed and in rare instances fulminant steatohepatitis occurred during the first postoperative year presumably as a result of the excessive weight loss following bypass surgery. 

 In patients with NAFLD/NASH with cirrhosis, bariatric surgery may be contraindicated in otherwise eligible obese subjects. Therefore, additional data are needed before recommending bariatric surgery as a treatment option for cirrhotic individuals with NASH [61, 78]. 

## 10. Statins

Due to a lack of evidence demonstrating that patients with NAFLD and NASH are at an increased risk for a drug-induced liver injury with the use of statins, statins have been used for the treatment of dyslipidemia in patients with both NAFLD and NASH. Statins however are not recommended specifically as a treatment of NASH independent of dyslipidemia.

## 11. Ursodeoxycholic Acid (UDCA)

Because of overwhelming lack of supportive data despite several trials, ursodeoxycholic acid is not recommended for the treatment of NAFLD or NASH. 

## 12. Other Drugs

Other drugs tested in trial-based settings in individuals with NAFLD/NASH include gemfibrozil, probucol, vitamin C, betaine, and N-acetylcysteine. To date, there is no convincing evidence for a beneficial effect of any of these agents.

## 13. Conclusions

NAFLD/NASH is a complex disease process wherein genetic factors and environmental influences combine to determine the disease phenotype and its progression. The relative importance of these various factors varies between populations depending on the presence or absence of specific modifier genes and lifestyle choices. Finally, physicians should be aware that patients with NASH and evidence of cirrhosis should be screened for the presence of esophageal and/or gastric varices as well as hepatocellular cancer (HCC). HCC can occur in the absence of cirrhosis in individuals with NAFLD/NASH. This fact influences the utilization of imaging studies in individuals with NAFLD/NASH. Exactly who to screen, when to begin screening, imaging methods, and how often it should be repeated remains to be determined.
